# Critically examining diversity in end-of-life family caregiving: implications for equitable caregiver support and Canada’s Compassionate Care Benefit

**DOI:** 10.1186/1475-9276-11-65

**Published:** 2012-11-01

**Authors:** Melissa Giesbrecht, Valorie A Crooks, Allison Williams, Olena Hankivsky

**Affiliations:** 1 Department of Geography, Simon Fraser University, Burnaby, BC, Canada; 2 School of Geography and Earth Sciences, McMaster University, Hamilton, ON, Canada; 3 School of Public Policy, Simon Fraser University, Vancouver, BC, Canada

**Keywords:** Diversity, Caregiving, Canada, End-of-life, Qualitative, Compassionate Care Benefit

## Abstract

**Introduction:**

Family (i.e., unpaid) caregiving has long been thought of as a ‘woman’s issue’, which ultimately results not only in gendered, but also financial and health inequities. Because of this, gender-based analyses have been prioritized in caregiving research. However, trends in current feminist scholarship demonstrate that gender intersects with other axes of difference, such as culture, socio-economic status, and geography to create diverse experiences. In this analysis we examine how formal front-line palliative care providers understand the role of such diversities in shaping Canadian family caregivers’ experiences of end-of-life care. In doing so we consider the implications of these findings for a social benefit program aimed at supporting family caregivers, namely the Compassionate Care Benefit (CCB).

**Methods:**

This analysis contributes to a utilization-focused evaluation of Canada’s CCB, a social program that provides job security and limited income assistance to Canadian family caregivers who take a temporary leave from employment to provide care for a dying family member at end-of-life. Fifty semi-structured phone interviews with front-line palliative care providers from across Canada were conducted and thematic diversity analysis of the transcripts ensued.

**Results:**

Findings reveal that experiences of caregiving are not homogenous and access to services and supports are not universal across Canada. Five axes of difference were commonly raised by front-line palliative care providers when discussing important differences in family caregivers’ experiences: culture, gender, geography, lifecourse stage, and material resources. Our findings reveal inequities with regard to accessing needed caregiver services and resources, including the CCB, based on these axes of difference.

**Conclusions:**

We contend that without considering diversity, patterns in vulnerability and inequity are overlooked, and thus continually reinforced in health policy. Based on our findings, we demonstrate that re-framing categorizations of caregivers can expose specific vulnerabilities and inequities while identifying implications for the CCB program as it is currently administered. From a policy perspective, this analysis demonstrates why diversity needs to be acknowledged in policy circles, including in relation to the CCB, and seeks to counteract single dimensional approaches for understanding caregiver needs at end-of-life. Such findings illustrate how diversity analysis can dramatically enhance evaluative health policy research.

## Background

Determining who provides care, whether paid or unpaid, is a complex and dynamic process embedded within socially- and politically- defined sets of expectations and practices regarding rights and responsibilities [1,2]. Among Western nations, neoliberal policies and resulting health care reforms are increasingly shifting this responsibility of care from the state to the voluntary and informal sectors [3]. This shift has resulted in an increased deinstitutionalization of care, moving caregiving out of formal settings like hospitals and into the community, especially the home. As a result, greater expectations are now being placed on those within the home, largely family members and/or friends who are unpaid and untrained, to take on the role of providing care in this informal environment [3-5]. Generally, these family caregivers^a^ provide physical and emotional care as well as care coordination, among other tasks, for those who are in need of support [6]. In Canada, family caregivers and the supports they provide have become the backbone of the health and long-term care systems [6], with estimates indicating that there are approximately 1.5-2 million family caregivers in Canada contributing up to $26 billion of unpaid care work annually [7,8]. Due to a rapidly aging population and unprecedented numbers of citizens requiring end-of-life care, a growing number of these caregivers are being called upon to provide care for dying family members [9]. Considering the valuable role that these family caregivers play in the Canadian health care system, it is imperative we seek ways to ensure they have access to the supports that they need to provide care in a way that does not negatively impact their own health and wellbeing.

Although family caregiving at end-of-life can bring positive, empowering, and memorable experiences [10], it is also commonly associated with personal costs. Importantly, such costs are not distributed equally across society. For example, the shift of care from institutions to the home means that those who work within the home, largely women, are filling the gaps in labour and services that have been left by neo-liberal policies [1,11]. Feminist scholars have long acknowledged that the role of family caregiving is largely taken up by women because they are often associated with the traditional gendered division of unpaid work within the home [1,12-14]. Emphasizing the gendered aspect of care provision, Bondi (2008) describes how caring work is ‘given’ to women and that this often becomes a defining characteristic of their self-identity and lifework. Taking a closer look, however, it becomes apparent that women are not one homogenous group, but are complex and diverse individuals who simultaneously inhabit other distinct socioeconomic, cultural, political, and historical locations, and as such, their experiences of caregiving are likely to vary dramatically [15,16]. Therefore, caregiving results not only in gendered inequities, but also complexly overlaps with other factors of social location that may result in particular economic and health inequities being experienced in light of giving care [13].

Taken together, the emotional, psychological, physical, and financial demands that occur as a result of family caregiving are commonly referred to as ‘caregiver burden’ [17]. For example, the mental drain associated with mastering vast amounts of new information on a range of complex issues (e.g., medication management, symptom observation) can be more than some caregivers are able to cope, thereby creating stress and ultimately burnout [18,19]. The negative health impacts associated with such burdens are significant and have been reported in older spousal caregivers to increase mortality rates by 63% when compared to non-caregivers [20]. Inequities in income are also commonly experienced by family caregivers. For example, the Canadian Caregiver Coalition (2009) reports that these caregivers frequently incur more than $100 per month in direct costs (e.g., supplies, prescriptions, travel costs), which totals approximately $80 million in out-of-pocket costs paid by Canadian caregivers each year. For some, the dual responsibility of maintaining paid employment while providing care is challenging and such stress can further diminish the health of family caregivers and heighten caregiver burden [9,19].

Importantly, the above-mentioned examples of negative health and economic impacts associated with caregiver burden are not experienced uniformly: inequities exist, which are shaped by vulnerability to stressors and exposure to risk [21]. Furthermore, family caregivers’ abilities to cope with stress and burden and to access needed supports is largely shaped by the situated social/physical locations in which they live [1,14,22], which in turn, influences whether or not they experience negative health outcomes.

Canada’s federal government responded to the need to lessen caregiver burden and better support the needs of family caregivers at end-of-life by implementing the Compassionate Care Benefit (CCB) program in January of 2004. The primary goal of the CCB is to alleviate financial burdens by providing income assistance and job security to workers who take temporary leave from employment to care for a terminally ill family member at risk of dying within 26 weeks [23]. Program recipients can receive up to 55% of their average insurable earnings, to a maximum of $485 per week, over a six-week period to provide care. Because Human Resources and Skills Development Canada (HRSDC) administers this program as an Employment Insurance special benefit, eligible applicants must demonstrate that their regular weekly earnings have decreased by more than 40% and that they have accumulated 600 insurable hours over the preceding 52-week period. Although estimates demonstrate that more than 1.5 million Canadians provide care for dying individuals annually [7], HRSDC reports that only 5,978 successful claims were made during the 2009/10 fiscal year [24]. One reason for the limited uptake is that those who are working part-time, are employed seasonally, or are unemployed are eliminated due to the eligibility criteria, thereby excluding many family caregivers. The design of the program itself is also thought to reinforce low uptake through lack of appeal to caregivers for a number of reasons, including that: support lasts for only six weeks, recipients must go through a two-week waiting period before benefits are initiated, and it is difficult to accurately prognosticate death within a 26-week window [25-27]. Aside from program-specific features, there exists a more critical obstacle to the CCB’s successful uptake: the general lack of public awareness regarding the existence of the program [28-30]. Specifically, family caregivers are routinely not gaining access to information about the CCB program.

Considering that family caregivers, and particularly end-of-life family caregivers, play such a significant role in Canadian society, it is imperative to seek strategies that minimize or alleviate inequitable caregiver burden and the potential negative physical/mental/emotional health, employment, and financial outcomes it bestows, especially upon those who are most vulnerable [6,9]. However, the diversity and differing vulnerabilities that exist within the caregiver population are rarely recognized in existing research and associated health and health-related policy. This signals the need for a re-framing of how we view caregivers and caregiving needs more generally, in order to develop effective policies and programs that recognize difference and account for inequities within this group. In relation to the CCB, what remains unexplored is how family caregivers’ differing social/physical locations may be informing the underutilization of the program, or may be exposing specific groups to harsher uptake barriers than others. In this article, we pose the question: for whom is this program *not* working? We address this question through undertaking a diversity analysis that highlights how particular axes of difference may ultimately inform family caregivers’ use of the CCB. More specifically, our objective is to examine family caregiving at the end-of-life in Canada from the perspective of formal front-line palliative care providers (e.g., community nurses, social workers) in order to gain a better understanding of the axes of difference directly impacting family caregivers’ support opportunities, access, and outcomes. Front-line palliative care providers’ employment allows them on-going access into the lived realities of numerous families experiencing death and dying. As such, they hold a broad and valuable experiential perspective from which to comment upon the general differences they observe between the family caregivers they interact with in their work. The results of our analysis are used to understand the implications of caregiver diversity for the CCB and also the need to re-frame how caregivers and caregiver needs are understood and acted upon in health and social policy more generally.

## Methods

This analysis contributes to a larger evaluation study that aimed to gather the perspectives of the CCB program’s key stakeholder groups, namely family caregivers, front-line palliative care providers, and human resources personnel, in order to offer policy-relevant recommendations for program improvement. The overarching methodology of the evaluative study is Patton’s [31] utilization-focused evaluative approach, which aims to inform program improvements through the use of research findings with a specific emphasis upon “*intended use by intended users*” [20,31], emphasis in original. Examining diversity within the caregiver experience was not an original objective of the CCB evaluation study, but rather emerged as an important issue from the evaluation study findings. Although family caregivers were interviewed for the larger evaluation, in this analysis we draw on interviews with front-line palliative care providers because they were able to ‘step-back’ from offering an experiential account of caregiving and comment more broadly on trends observed among those family caregivers they have worked with. These observations were informed by their employment, which offers them intimate access to lived realities of families experiencing death and dying.

### Data collection

Fifty front-line palliative care providers were sought to participate in phone interviews from across Canada, ten from each of five provinces chosen to reflect Canada’s linguistic and regional diversity: British Columbia, Manitoba, Ontario, Quebec, and Newfoundland and Labrador. We purposely aimed to include participants from an array of workplace settings (e.g., administrative, clinical, home care) and occupational groups (e.g., nurses, physicians, social workers) in order to garner as much diversity in employment sectors as possible. Participants were required to be formal palliative care providers working in one of the target provinces whose employment placed them in *direct* contact with end-of-life family caregivers and/or care recipients. Prior to data collection, ethics approval was granted by the research ethics offices at Simon Fraser and McMaster Universities.

The recruitment strategy involved widely disseminating an information letter written in English and French that summarized the study purpose and participant inclusion criteria. The letter was sent to a number of palliative and hospice organizations in the target provinces asking them to share it with their own employees and other organizations in their networks. Interested potential participants were asked to reply by e-mail or call a toll-free number to schedule an interview in either English or French at a time convenient for them.

Semi-structured interviews were conducted via telephone by the first author. Generally, the interviews lasted 30 minutes and inquired into: the CCB’s usefulness; its barriers and facilitators to access; experiences of recommending the CCB to potential applicants; and suggestions for program improvement. Prior to the interview, interviewees were informed of their rights as participants in a research study and provided their verbal consent. In total, 48 English- and two French-language interviews were conducted (*n* = 50), which resulted in 10 participants for each of the five provinces. Participants came from a variety of occupational groupings and work settings, as shown in Table 1. Thirty-seven held full-time employment, 12 worked part-time, and one had retired one month prior to the interview. Participants’ years of experience working in palliative care are shown in Table 2.

**Table 1:** Front-line palliative care provider participants by occupational grouping

**Occupation**	**Number of participants**
Social Worker	11
Palliative Care Director / Coordinator	9
Clinical/Oncology/Palliative Nurse	7
Community Health/Home Care Nurse	7
Oncologist/Physician	6
Chaplain/ Pastoral Care	2
Counselor	2
Volunteer Coordinator	2
Facility Patient Care Manager	1
National Nursing Officer	1
Nurse Coordinator	1
Occupational Therapist (Home Care)	1
*TOTAL*	*50*

**Table 2:** Employment experience of the front-line palliative care provider participants

**Years of employment experience in palliative care**	**Number of participants**
Less than 1 year	5
1 to 5 years	16
6 to 10 years	12
11 to 15 years	5
16 to 20 years	5
Over 20 years	6
*Total*	*50*

### Analysis

Forty-nine interviews were digitally recorded and transcribed verbatim, and one was recorded through note taking due to this participant’s preference to not be recorded. Transcripts and notes were entered into NVivo7™ data management software and thematic analysis was conducted. Thematic analysis involves identifying dominant emergent themes in the data that are then used as categories for analysis [32]. Emerging from the larger evaluative study findings was the main theme of ‘diversity’ among caregivers and caregiving experiences. Thus, a secondary analysis was performed upon the front-line palliative care provider data set using a critical diversity method.

To enact a critical diversity analysis we first developed a coding scheme that integrated both inductive and deductive perspectives informed by Hankivsky, et al.’s [33] definitions of social categories. Our six-step process of coding involved: (1) reviewing three randomly selected transcripts to identify initial themes regarding diversity; (2) drafting a full coding scheme; (3) revising the full coding scheme following further transcript review for confirmation; (4) coding five transcripts; (5) reviewing coded transcripts in order to refine the scheme (e.g., collapsing redundant themes); and (6) coding the entire dataset with the refined scheme. In order to enhance consistency of interpretation, multiple investigators were involved in implementing the six-step coding process, as well as in reviewing the associated coding extracts that were used to inform the present analysis.

Our critical diversity analysis draws upon the emergent inductive codes of ‘culture’, ‘gender’, and ‘geography’, among others, as well as a number of deductive sub-codes such as ‘family caregivers – differences in – culture’ and ‘family caregivers – differences in – gender’. Reviewing these particular coding extracts, we determined as a group the scope, limitations, and interrelationships within and between each axis of diversity, creating an interpretive framework for understanding how each was understood by the interviewees. Our approach to doing this was informed by Young’s notion of seriality [34], which disrupts the notion that ‘groups’ are to be organized by single-dimensional characteristics (e.g., women), and emphasizes that people are individuals in a ‘series’ with their positioning based on various sets of material and immaterial social constructs. In addition to this, our analysis was also informed by the intersectionality work of Hankivsky et al. [33], which requires consideration of simultaneous interactions between different aspects of social identity as well as the impact of systems and processes of oppression and domination. Our approach of intersectionality used in this analysis is grounded in lived experiences, while providing a theoretical foundation for the pursuit of social justice. Importantly, we did not begin with predetermined categories of difference that were of interest to us, but instead relied on these axes to emerge from the data through undertaking the coding and analyses processes. Following our consensus regarding the interpretation of various axes of difference that emerged, we moved to identify ways in which these particular diversities matter for the CCB specifically and other caregiver support programs more broadly.

## Results

Thematic critical diversity analysis revealed five axes of difference that were commonly raised by front-line palliative care providers when discussing end-of-life family caregivers: (1) culture; (2) gender; (3) geography; (4) lifecourse stage; and (5) material resources. While there is no doubt that other significant differences exist among family caregivers that directly influence their experiences of providing care, such as sexual orientation, (dis)ability, and health status, they were not explicitly discussed by the participants and so are not examined here. It is important to emphasize our recognition that such axes of difference are not static containers, but are fluid and dynamic, varying across time, place, and especially context [35,36]. Furthermore, we also recognize that lived realities are highly complex and ‘differences’ are inherently constructed, relational, and interconnected [16,37]. However, we believe that a critical starting point to addressing inequities lies in determining *what* differences exist and *how* they impact experiences of family caregiving at end-of-life. As such, in the following subsections we discuss our findings of each of the five axes of difference, which are defined in Table 3 in detail. In the discussion section we then move to consider these axes in relation to one another, and how their intersection may heighten the barriers family caregivers face in utilizing the CCB program. 

**Table 3:** Operating definitions employed in the diversity analysis

**Axis of difference**	**Operating definition**
Culture	the totality of the ideas, beliefs, values, knowledge, and way of life of a group of people who share certain historical, religious, racial, linguistic, ethnic and/or social backgrounds
Gender	the manner in which a society defines and constrains the array of socially constructed roles and relationships, personality traits, attitudes, behaviours, values, relative power and influences based on a differential basis of being a ‘woman’ or ‘man’
Geography	the physical and social places in which various activities happen that are shaped by actions, processes, and other powerful happenings occurring both within and beyond them
Lifecourse Stage	the sequence of socially defined events and roles that individuals enact over the progression of their life from birth to death
Material Resources	the tangible goods and consumables and the means by which they are purchased, wherein an absence of these resources can result in material deprivation

Operating definitions informed by Hankivsky et al. [33].

### Culture

Our findings indicate that front-line palliative care providers perceive cultural differences to play a major role in influencing experiences of family caregiving at end-of-life, especially when personal beliefs contrast with the clinical culture of the Canadian health care system. For example, an occupational therapist noted that in her region “*a lot of our doctors are not from the area or even from Canada, so I guess the biggest cultural barrier is between the doctors and the patients themselves.*” Participants discussed how families from various cultural groups can have differing understandings, priorities, and/or needs, thus requiring additional support in order to achieve quality end-of-life care. As an example, a palliative care coordinator explained that she had worked with a family of Chinese heritage who did not want a death to occur in their home as this was believed to negatively affect the value of the home, both spiritually and financially. In this case, cultural preferences had informed decisions regarding the place of care and ultimately death.

Generally, participants believed that caregiver supports (e.g., psychosocial, religious, spiritual, bereavement) needed to accommodate families from differing cultural backgrounds as much as possible. Emphasizing the complexity of this task, however, a palliative care nurse remarked “[t]*here’s lots to recognizing the different cultures and how different people approach dying, how they want their family members to approach it.* [But] *do they* [care recipients and family caregivers] *ever want to talk about it?”* Lack of discussion about cultural needs may result in some family caregivers not having access to needed supports. For example, several participants explained that First Nations or Métis family caregivers and care recipients should always be asked if they have any spiritual and cultural needs related to end-of-life or family caregiving, such as performing a sweet grass or smudging ceremony^b^. The challenge here, however, is that front-line providers must first be able to discern which families are First Nation or Métis in order to ask them if they would like such supports. One’s cultural heritage, however, may not always be easily recognizable; therefore, such an approach relies heavily on self-identification (i.e., explicitly presenting oneself as First Nations or Métis to others). As self-identification may not always be appropriate or desirable, First Nation or Métis may face barriers in accessing supports that meet their cultural needs.

As per the definition of culture used for this analysis, language is one of the many various cultural components discussed by participants. Participants raised language as an important issue in the experience of caregiving, specifically with regard to language barriers and caregivers’ abilities to access necessary information and supports. These comments were often raised with regard to newcomers to Canada, where participants stressed that not being able to communicate is a major barrier to determining caregiver needs. A social worker explained that: “*the challenge sometimes is getting someone who speaks English* [in the home]. *And sometimes the ones who do speak English are working, while it’s the sister-in-law or the daughter-in-law, the one that’s providing all the care, that doesn’t speak English, so we use translators a lot.”* However, communication through a translator was seen as problematic, especially if information was being *“filtered”* through another family member because details may become exaggerated upon or simply left out. It was also noted that language barriers can create major informational needs and thus increase the risk of caregiver burnout and stress as these caregivers can be hesitant to seek out the help they need.

### Gender

Traditionally, and still today, the role of family caregiver is largely ascribed to women. Unsurprisingly, participants confirmed this as a clear observation from their work experience^c^. Some participants, however, stated that they have noticed a recent increase in men taking on caregiving responsibilities, though not necessarily as the primary caregiver. As a social worker explained, *“*[t]*he reality is that there is a gender bias still in our society, so women are still primarily the family caregivers, they’re primarily the child-rearers. There are many, many men who are doing those things, but women are still primarily in that role.”* Participants widely agreed with this view, explaining that they perceived societal expectations to still fall more heavily upon women to provide care within the home, thus resulting in the majority of the family caregivers they interacted with being women. In some situations, participants also observed women who were not immediate family, for example a daughter-in-law or sister-in-law, providing care, which further demonstrates the extent of gendered implications associated with caregiving expectations.

Although it was clear that participants were cautious to convey gendered generalizations, some did believe that differences existed between men and women with regard to caregiving styles. For example, this community health care nurse explained that:

"Female caregivers are more in tune to the person’s physical needs, whereas male caregivers tend to get very organized and business-like about it. You know, they’ll pull together little flow-charts and books and sort of stand back and let me deal with the things like bowel care and hygiene… I find that men have a really hard time with that personal care aspect."

Given such observations, it is not surprising that some participants raised gender-specific caregiving needs. These participants felt that it was men, rather than women, who required extra support in order to successfully fulfill their role as a family caregiver. For example, a palliative care coordinator stated that in some cases, challenges arise when a woman who has always taken care of everything in the home is dying and in need of care “*and the husband doesn’t have a clue how to, you know, do anything*…*”* This sentiment was echoed by a home care nurse who stated that sometimes “[m]*en*…*looking after women, where the woman has been the manager of the house, need a lot more information on managing home situations than a woman might.”* Considering these findings, it becomes apparent that gender and gendered expectations regarding behaviour play a role in determining caregiver support needs.

### Geography

Several front-line palliative care providers discussed the impact that geographic differences have on the experience of family caregiving at end-of-life. Specifically, they felt that where one lives determines access to services. The most prominent differences were raised by participants working in Newfoundland and Labrador^d^ who believed that the relatively isolated location of this lightly populated province created unique challenges for families in terms of accessing end-of-life care supports compared to the rest of Canada. Several participants from this province explained that a rapidly aging population, in conjunction with the increased outmigration of the youth, has left few able bodied family caregivers to draw from for support. As an oncology nurse remarked:

"[t]he situations in our province, they [caregivers] thin out quite a bit because of migrations, and smaller family size and that kind of stuff. We have a lot of people living in smaller areas who really have nobody around them now. Or the people around them are very elderly and no better off themselves, or able to care for the person who's dying."

Those participants working in rural communities throughout Canada also identified unique challenges for caregivers associated with low populations residing across vast distances, which results in fewer resources being made available and long commutes to access needed supports. Participants from rural communities also commented on the extra costs family caregivers from these areas must endure in order to travel to-and-from urban centers to access supplies and services.

Another geographic difference that emerged from the interviews involves the place where care and ultimately death occurs, such as the hospital, hospice, or home. According to participants, the preferred place of care and death was said to differ according to each family’s wishes, though generally family caregivers preferred to have the care recipient stay at home for as long as possible. Regardless of these preferences, it was noted that decisions regarding the location of care were almost always made based on the level of access to supports and the resources caregivers had to draw from. However, because of a lack of access to needed supports and resources within the home, participants felt that some family caregivers are left with no option but to move care recipients to formal settings such as a hospital palliative unit. Especially approaching the very end-of-life, participants expressed that continuous support is required and thus family caregivers who do not have resources or access to supports will need to cease providing care in the home. A palliative care coordinator explained that *‘…as a healthcare professional…I think there would be a lot more caregiving going on in the homes if we could support more people to caregive for their family.”* As such, one’s geographic location in relation to supports and services plays a critical role in enabling care provision in certain environments, such as the home.

### Lifecourse stage

Participants made a number of comments indicating that where a caregiver was situated in his/her lifecourse, versus her/his specific age, significantly impacted the types of supports required by family caregivers at end-of-life^e^. Participants stated that care recipients are generally elderly, over the age of 80, and that it was common to find spouses providing end-of-life care, resulting in what one nurse explained as “*seniors taking care of seniors.*” Explicitly commenting on the differences in stages of the lifecourse among family caregivers, one social worker stated that:

"…if they [caregivers] are seniors…you’re going to be dealing with perhaps a caregiver who has health problems too and so may not have the physical stamina or ability to give intensive care…if the person [care recipient] has a high care need, it may be overwhelming to the spouse."

On the other hand, it was sometimes mentioned that because elderly spousal caregivers are typically retired or career homemakers, they are viewed as ideally situated to provide care because there will be no disruption to employment or income levels.

Although many end-of-life family caregivers were thought to be elderly, several participants explained that it is not uncommon for children to take on the role of caregiving for dying parents. This was thought to be concerning if the daughter/son caregiver also had a family with young children of their own to care for. It was explained that family caregivers who find themselves in this ‘sandwich generation’ are likely to experience conflicting familial roles, which results in particular challenges, stresses, and support needs, such as child care. Concerns were also raised regarding family caregivers from younger families where one spouse is at the end-of-life and the other is providing their care. Again, participants explained that this scenario is incredibly challenging for families where young children are involved. A broad concern regarding these lifecourse-related scenarios is that younger families were thought to be more vulnerable financially than older ones: “*…with a younger family, if one of the spouses is the one who’s dying and is unable to work, and it has been a two income family, that’s a huge impact on the family if they’re losing one income.”* As a result, younger families are said to require more support in terms of financial and job security: *“Especially our young families, they need to know that they are going to have job security, and resources, financial resources for the time period that they’re going to be off* [from paid employment to provide care].” The provision of such security is muddied by the fact that caregiving at end-of-life rarely follows a predictable trajectory.

### Material resources

Although ‘socio-economic status’ is an axis of difference often highlighted in diversity analyses, in this study participants’ comments pertained mostly to the specific circumstance of access to material resources rather than the broader category of socio-economic status. Many front-line palliative care providers emphasized how variations in families’ access to material resources, such as income, equipment, medication, and formal respite and home care support, resulted in dramatic differences in the caregiving experience. As one social worker said, “*we say that homecare is universal* [in Canada]*, but it’s not really universal. It’s based on your finances and what you’re able to provide in terms of concrete help…”* There are many extra financial costs associated with providing care for a dying family member in the home, such as making home renovations and purchasing, renting, and/or installing medical equipment. A palliative home care nurse commented on these costs:

"I think that caring for someone in their own home is expensive. And I don’t think we look into that enough, because…they’ve [caregiver] taken time off from work, they don’t have an income, and then they’ve got all these extra things that they have to get. They have to buy a walker; they have to get a wheelchair – none of that is something that we provide."

Participants were particularly concerned about families who do not have private medical insurance and therefore are required to pay in full for needed supplies and other items, which in some cases places families in great financial stress. An oncology nurse shared one of her experiences of working with a family that experienced financial hardship as a result of caregiving responsibilities:

"…I saw that disease destroy, financially ruin, people. Because before they were diagnosed with the disease, they had a bit of money. They were…middle class people with a little bit of money in the bank. And by the time the person affected with the disease ended up dying, the family had nothing left… When the person died, they couldn’t afford to take the body home…and that was the only time they received a bit of help, was when they had to go to social services to get the body home. It’s devastating."

Participants pointed out that financial pressure may place increased stress on family caregivers, thereby negatively affecting their health.

Some family caregivers’ inabilities to access material resources, particularly medications, respite care, and transportation, results in inequitable care outcomes. For example, a palliative care coordinator said that “*quite often patients are suffering because they* [caregivers] *don’t have the money to buy medications*.” Such a situation may not only be disheartening for the care recipient, but also the caregiver who is unable to manage pain and relieve distress. Access to in-home respite support was also believed by many participants to be a resource that greatly affected caregivers’ abilities to manage their role through mitigating the risk of caregiver burnout. With regard to family caregivers’ need for respite support, a social worker stated that *“if we’re sending people* [care recipients] *home with the expectation that they’re having 24 hour care, it’s only realistic if that person* [family caregiver] *gets some time to breathe as well.”* Many participants commented on how unavailable this support generally is for Canadians, not only due to costs, but also to geographic issues whereby in many rural and remote areas respite support is simply not an option. Furthermore, access to transportation was discussed as being a material necessity for family caregivers in order to take care-recipients to appointments. However, not all caregivers have reasonable access to transportation, let alone a vehicle that can accommodate the space and comfort requirements of a care recipient.

## Discussion

Our analysis of 50 interviews with front-line palliative care providers reveals that acknowledging diversity among Canadian family caregivers is an important aspect of understanding the caregiving experience. Apparent is that front-line palliative care providers observed cultural, gendered, geographic, lifecourse stage, and material differences between family caregivers, shaping the types of experiences caregivers have as well as the supports they have access to. These axes of difference also reveal segments of the caregiver population that may be particularly vulnerable to experiencing inequities with regard to accessing needed services and resources. These groups include non-English or non-French-speakers, cultural minorities, rural residents, employed caregivers who are women, caregivers who are men, caregivers with young children, and those who do not have or are unable to purchase meaningful material supports. Given that having adequate access to services and resources serves to lessen exposure to caregiver burden [38,39], it is quite likely that these groups also have increased risk of negative health outcomes as a result of taking on a caregiving role. Furthermore, vulnerability to caregiver stress, burden, and negative health outcomes may be amplified for those whose lived reality overlaps *multiple* segments of these particular population groups. Without considering diversity, such patterns in vulnerabilities and inequities would simply be overlooked, and ultimately, continually reinforced [33].

### Implications for the CCB

From a policy perspective, this analysis demonstrates why diversity needs to be acknowledged in policy circles, including in relation to the CCB, and seeks to counteract single dimensional approaches for understanding family caregiver needs at end-of-life that simply cannot account for inequities. As noted above, the objective of the CCB program is to provide employment security with basic financial assistance for eligible family caregivers during the last eight weeks of a care recipient’s life. A number of implications emerge from the findings of this critical diversity analysis for the structure of the program and the way in which it is administered. Importantly, in reviewing these implications, summarized in Table 4, it becomes clear that the solutions for improving supports for end-of-life family caregivers in Canada do not rest solely on adjusting the CCB program. Due to the complexity of death and dying, there is a need for multiple governmental sectors (e.g., Medicare, employment insurance, family allowance) to become involved in better supporting end-of-life family caregivers at multiple levels (e.g., local, provincial, and national) in order to address the complex needs of families experiencing death and dying. This multi-sectoral approach, however, requires coordination, a shared vision, and political commitment from leaders and champions in order to be successful [40]. 

**Table 4:** Implications for the CCB program

**Axis of difference**	**CCB implications**
Culture	*Language:* Limited access to information on CCB outside of English and French
	*(New) Immigrants:* Linguistic and cultural barriers may limit caregivers’ abilities to complete applications forms or front-line providers’ abilities to convey program information
Gender	*Eligibility:* Women are more likely to be ineligible for the CCB due to employment circumstances while more likely to serve as family caregivers
	*Utilization:* Men are proportionally underrepresented among successful CCB applicants, which suggests that the program may not meet their needs
Geography	*Travel:* Costs for travel, local or otherwise, to provide care are not covered by the CCB program
	*Place of Care and Death:* Lack of formal support may discourage potential or on-going family caregivers from providing care in the home when receiving the CCB
Lifecourse Stage	*Elderly:* Retired caregivers are not eligible for the CCB program
	*Young Families:* Costs for child care support are not covered by the CCB program
Material Resources	*Homecare:* High costs to provide care in the home are not covered by the CCB program
	*Supply Costs:* High costs of services and supplies for caregivers without medical insurance are not covered by the CCB

The CCB is commonly thought of as a positive step towards broadly meeting the needs of Canada’s end-of-life family caregivers [26]. However, this analysis has shown that individuals who fall within this broad demographic are likely to experience barriers to accessing this support. For example, though Canada is renowned for its multicultural landscape [41], formal (i.e., government-sponsored) information about the CCB and its application forms are only available in English and French [23]. This leaves front-line palliative care providers and community groups to play a significant role in informing groups such as new immigrants and linguistic minorities about the program through websites and fact-sheets. However, interviewees clearly pointed out that informational access to the CCB can be hindered if front-line providers are unable to communicate well enough with family caregivers to assess their needs, which may result in them not informing families of the CCB’s existence or assisting them with completing the application form. Here, cultural brokers and translators may be able to play a valuable role.

As we have noted above, research has repeatedly demonstrated that women are most likely to become family caregivers at end-of-life in Canada; however, it is also women who are least likely to be eligible for the CCB. In Canada, women generally make up the majority of stay-at-home parents and part-time workers and thus are less likely than men to contribute to Employment Insurance and be able to draw upon its programs [27]. Although we might expect women to have lower uptake of the CCB, program utilization data show that they do indeed make up the majority of CCB claimants [30]. These same data also show that women receive on average lower weekly benefit payments than men [42], which is a direct result of women applicants having more limited salaries. These utilization data point to an interesting set of paradoxes: while women are generally less likely than men to be eligible for the CCB due to having more limited labour market participation, they are actually more likely than men to receive the Benefit; and, while men are likely to receive greater financial support while on the CCB- due to higher salaries, they are less likely than women to actually use the program and thus may be underutilizing the Benefit relative to their labour market participation. Such circumstances create clear implications for the CCB, the solutions for which extend well beyond the scope of the program.

The interviews revealed that geography, particularly differences in access to services and the presence of family caregivers between places, is a significant axis of difference in the caregiving experience. For example, interviewees reported that Newfoundland and Labrador’s rapidly aging population and concurrent high rates of youth out-migration has generally resulted in the elderly caregiving for the elderly. This demographic trend has been well established in statistical reports [43]. Although this region is in great need of caregiver support, the CCB as it is currently administered does not cover nor supplement the cost of travel for family members to relocate for care provision. Furthermore, at a more localized level, travel within or between communities to gather supplies and access medical appointments is also not covered by the program. Such realities may require family caregivers in these locations to use CCB program monies to offset travel costs.

Participants viewed one’s lifecourse stage to greatly impact the caregiving experience, including a caregiver’s need for particular types of support such as the CCB. Importantly, participants commented that younger “*sandwich generation*” caregivers are more likely to be participating in the workforce while providing care, thus heightening their financial vulnerability. As such, the CCB may be best suited for meeting some of these caregivers’ needs by ensuring their jobs are secured while providing some financial assistance. Participants also explained that it was common for younger caregivers to need access to child care while providing end-of-life family caregiving. However, the CCB does not provide a child care allowance, which may result in some relying on the financial assistance of the CCB to cover child care costs rather than truly supplementing income. Because the CCB does not consider circumstances regarding the loss of dual incomes, or the shifting of child care responsibilities due to caregiving demands, working-aged family caregivers may not find the CCB to be a viable option to meet their financial needs.

Finally, many participants emphasized how variations in access to material resources, such as medication, equipment, and respite care, result in noticeable differences in the caregiving experience. Access to such resources was thought to impact caregivers’ quality of life as well as decisions regarding where care should take place. As it is currently administered, the CCB provides relatively limited financial assistance (up to a maximum of $485 per week). This minimal level of income support has been viewed by many as major deterrent for caregivers to utilize the CCB [25,27,44]. The financial costs associated with purchasing equipment, supplies, and medications can be relatively high for some caregivers, and the financial assistance provided by the CCB may not be meeting their financial support needs. Moreover, it has been found that caregiving can potentially enhance the risk of poverty as it contributes to high levels of stress and associated negative health outcomes, which in turn affects caregivers’ abilities to return to paid employment [45].

### Intersecting differences – a future research direction

It is important to explicitly recognize that every caregiving situation is different and that every caregiver has unique concerns and difficulties [46]. However, dominant approaches to caregiving research and policy to date have failed to adequately acknowledge issues of diversity or what diversity in the experiences of caregivers might mean for existing programs and/or services, or for advancement in policy. In this research, we have moved beyond many other studies by explicitly teasing apart the axes of difference reported on by participants and organizing them into separate categories for the purpose of conducting a critical diversity analysis. Advancing feminist thought, and keeping in line with intersectionality scholarship, however, we do recognize that these axes of difference are inherently linked, which in turn structurally shape one’s social/physical location and thus influence one’s caregiving experience. Reflecting this complexity, intersectional scholars (for example, see [36,47,48]) observe that no single dimension of diversity or difference should be given favour but that, instead, researchers should consider simultaneous interactions between these dimensions [47-49]. In other words, it may not be a caregiver’s experience as a woman that exposes her to the most significant inequities, even though this axis tends to receive the most attention in the caregiving literature [13-16]. Rather, it may be her collective inability to speak English or French, residence in a rural community, and lack of access to appropriate medical equipment *intersecting with* gender that determine support needs and in particular, whether or not programs like the CCB are effective in meeting them. Following from the current analysis, there is a need for caregiving research to examine and articulate such intersections among axes of difference in order to adequately consider and address existing inequities as well as the underlying structures of power that reinforce them.

Our findings shed light upon some major differences that exist among family caregivers that can dramatically shape caregiving experiences and access to meaningful supports. However, this is simply the first step and this analysis serves to signal the need to further pursue this line of inquiry, including the application of social justice approaches, in future caregiving research and policy creation. Attention to diversity and inequity is slowly beginning to emerge in the caregiving literature (see [50-55]). For example, in their examination of foreign domestic care workers, Hsuing and Nichol [53] argue that the complexity of the experiences of these workers “cannot be fully captured simply by examining any single axis of their identity; it requires an examination of the intersections of race, class, and gender” (p.773). To the best of our knowledge, intersectionality has yet to be applied to the context of end-of-life caregiving. Based on our findings and the traction they hold for more general examinations of care work, an intersectional analysis shows great promise in advancing knowledge in relation to end-of-life caregiving, and in the longer term may provide evidence that will be the basis of much needed critical challenges to Canadian policy in this area, as well as policies in other countries that rely heavily on the labour of unpaid family caregivers.

### Limitations

This study has three main limitations. First, we use the perspectives of front-line palliative care providers to draw conclusions about family caregivers. Although this was done purposely because front-line palliative care providers have exposure to a range of family caregivers and can comment on this, the analysis misses out on the experiential comments that can be offered by family caregivers themselves. This serves as an important direction for future research. Second, our reliance on phone interviewing means that we were unable to observe nuances of facial expression and other subtleties that in-person interviewing allows. However, we used phone interviews because they are cost effective, particularly given our cross-country sample, and are known to yield reliable data [56,57] and so we are not concerned that this limitation has had a negative impact on the analysis. Third, as there is no population- or national-level data that characterizes the full spectrum of front-line palliative care providers, we cannot know how representative our participants are of this health worker group. As our study is qualitative in nature, we do not actually seek representativeness to achieve overall generalizability, but rather the transferability of the findings. Given this, the lack of population- or national-level data did not serve as a true limitation in our research.

## Conclusions

Through conducting a critical diversity analysis, a nuanced portrait of the complex realities experienced by Canadian family caregivers within the context of end-of-life care has been revealed in this article. It is important to note, however, that this analysis considers front-line palliative care providers’ perspectives, and not those of the family caregivers themselves, and so should be taken as such. Yet, front-line palliative care providers have valuable perspectives regarding the diversity of families experiencing death and dying and therefore should be considered in policy-related caregiving research. While most research in this field does not explicitly consider diversity, that which does tends to focus on one or two pre-defined axes, such as gender and/or economic status and/or culture [21,58,59]. While the findings of this study point to the importance of these axes, they also demonstrate that others are also of great significance in shaping the caregiving experience, such as geography and lifecourse stage. We see these findings as a critical first step in exploring what meaningful differences exist among family caregivers and how these differences impact caregiving experiences at end-of-life. We believe that understanding these meaningful differences can inform the development of more effective and equitable policies and supports.

In this analysis we have helped to disrupt the common policy discourse that implies family caregiving is simply a gendered experience. In doing so we have provided input that not only can inform CCB improvement, but can also provide valuable insights on how policy-makers can most equitably meet the needs of family caregivers in Canada. Importantly, our findings also signal the need for a *re-framing* on how we view and categorize family caregivers and understand their needs. This involves recognizing that some groups of caregivers may be particularly vulnerable to caregiver burden or other negative health outcomes in addition to barriers to accessing needed supports. Finally, this analysis has helped to cast light upon the often invisible work of and hidden burdens experienced by family caregivers. Their effort, understanding, and compassion enables so many dying individuals to live out their final days with dignity [6]. It is thus imperative for us to recognize the extraordinary effort that is made every day by family caregivers who care for dying individuals with dedication and ensure that they are provided with all the means necessary to carry out this valuable work.

## Endnotes

^a^In this paper, we use the term family caregiver to refer to those family members, friends, and/or close others who informally provide care to a recipient, often without out pay.

^b^Smudging ceremonies involve the burning of clipped herbs, such as sweet grass, sage, or cedar to create a smoke that can be lightly brushed over one’s body. This is done to cleanse one, both spiritually and physically, of any bad spirits or negative energy.

^c^An analysis that offers further discussion on some of the geographic and gender differences highlighted in this article can be found in Giesbrecht et al. 2010.

^d^Newfoundland and Labrador is a province of Canada located on the Atlantic Coast.

^e^Although age is often related to one’s experiences at various stages of the lifecourse, social experiences are not biologically determined by age, hence our decision to use lifecourse for this axis of difference.

## Abbreviations

CCB: Compassionate Care Benefit; HRSDC: Human resources and skills development Canada.

## Competing interests

The authors declare that they have no competing interests.

## Authors’ contributions

MG conducted the participant interviews, led the thematic diversity analysis, and drafted the manuscript with ongoing input from VAC. VAC and AW participated in the design of the study and the thematic diversity analysis. OH participated in the thematic diversity analysis. All authors read, made suggested revisions to, and approved the final manuscript.

## Authors’ information

**MG** is training as a health geographer and is a PhD Candidate in the Department of Geography at Simon Fraser University (SFU), Canada. Her research focuses on exploring diversity among users of palliative care services/supports and identifying how ‘place’ intersects with subject positions to shape access, end-of-life experiences, and palliative care outcomes.

**VAC** is an Associate Professor in the Department of Geography at SFU. Trained as a health geographer, she primarily uses qualitative methods to examine issues pertaining to access to and use of health services.

**AW** is trained as a social geographer, specializing in health research addressing: health care services, quality of life, family caregiving, critical policy/program evaluation, and therapeutic landscapes. She currently is an Associate Professor at McMaster University (Canada).

**OH** is an Associate Professor in the School of Public Policy at SFU. She is the Director of the Institute for Intersectionality Research and Policy at SFU. She specializes in public policy and political theory and is an internationally recognized expert in gender mainstreaming, gender based analysis, health determinants, and intersectionality-based analysis.
